# Potential Use of Hyperbaric Oxygen Therapy in Orthodontic Treatment: A Systematic Review of Animal Studies

**DOI:** 10.1055/s-0042-1755625

**Published:** 2022-10-11

**Authors:** Yun Mukmin Akbar, Ani Melani Maskoen, Endah Mardiati, Ganesha Wandawa, Arlette Suzy Setiawan

**Affiliations:** 1 Doctoral Program, Faculty of Dentistry, Universitas Padjadjaran, Bandung, Indonesia; 2 Department of Research Development, Indonesian Naval Dental Institute R.E. Martadinata, Jakarta, Indonesia; 3 Department of Oral Biology, Faculty of Dentistry, Universitas Padjadjaran, Bandung, Indonesia; 4 Department of Orthodontics, Faculty of Dentistry, Universitas Padjadjaran, Bandung, Indonesia; 5 Department of Pediatric Dentistry, Faculty of Dentistry, Universitas Padjadjaran, Bandung, Indonesia

**Keywords:** orthodontic treatment, orthodontic tooth movement, hyperbaric oxygen therapy, HBOT, animal studies

## Abstract

Understanding the fundamental principles of tooth movement could reduce the duration of treatment and achieve a stable outcome, resulting in patient satisfaction. Hyperbaric oxygen therapy was a modality in which a patient inhaled 100% O _2_ while subjected to high atmospheric pressure. Hyperbaric oxygen therapy facilitated the supply of oxygen to the human body's organs and tissues and served a variety of applications, including patient care and wound treatment. This review article aimed to describe animal studies of the potential effects of hyperbaric oxygen therapy in orthodontic therapy. It was conducted using a systematic literature review method, including searching PubMed and Google Scholar for publications relevant to the research topics. The search was filtered to include only research on orthodontic treatment and hyperbaric oxygen therapy and was published in any year. Articles that did not specify biological components of orthodontic tooth movement (OTM) were excluded. The Preferred Reporting Items identified the papers for the Systematic Reviews and Meta-Analyses (PRISMA) strategy, which resulted in the selection of 11 publications. Hyperbaric oxygen therapy affected parameters of biomarkers representing the clinical, molecular, and cellular biology of bone formation and resorption in periodontal tissues in responding to orthodontic physical forces, including alkaline phosphatase, collagen synthesis, osteoblast, osteoclast, osteocyte, type I collagen, vascular endothelial growth factor, osteocalcin, fibroblast, matrix metalloproteinase-8, transforming growth factor-β, partial pressure of oxygen, partial pressure of carbon dioxide, trabecular bone density, and tooth mobility. Hyperbaric oxygen therapy induced an inflammatory response to follow OTM events during active orthodontic therapy. Hyperbaric oxygen therapy might play a role in the tissue healing process during passive treatment. Nonetheless, additional research should be conducted to establish the efficacy of hyperbaric oxygen therapy in orthodontics.

## Introduction

Orthodontic treatment is predicated on the idea that applying pressure on a tooth would induce it to migrate as the surrounding bone reformed. The bone is deliberately removed from some areas and added to the others. The tooth migrated through the bone, bringing the periodontal ligament (PDL) with it. Since the PDL mediated the bony reaction, tooth movement was primarily a PDL event. 1 Understanding the basic concept of tooth movement might help shorten treatment times, stabilize the result, and increase patient satisfaction. 2 An optimum orthodontic force maximizes cellular responsiveness and tissue stability, whereas an unfavorable one might trigger adverse tissue reactions. Tooth movement is induced by an aseptic inflammation associated with histological findings of necrotic/hyaline sites, vasodilation, and leukocyte migration from blood vessels. 3 Undifferentiated mesenchymal cells and their progeny in fibroblasts and osteoblasts accounted for most of the PDL cellular components. During normal function, the ligament's collagen was constantly rebuilt and regenerated. The same cells could act as fibroblasts and fibroclasts, producing new collagenous matrix components and degrading previously made collagen. 1

Hyperbaric oxygen therapy (HBOT) is a procedure in which a patient inhales 100% oxygen while being subjected to an increased atmospheric pressure up to 2 to 3 atmospheres absolute for 1.5 to 2 hours, one to three times per day, depending on the indication. 4 5 6 7 The intracellular genesis of reactive oxygen and nitrogen species was a critical mechanism for HBOT. The role of reactive substances such as oxygen and nitrogen in this process is revealed through cell signaling transduction cascades. Nitrogen and reactive oxygen species combined cause cell damage, resulting in nitrative stress. 8 HBOT has facilitated the delivery of oxygen to the tissues; as a result, wound healing might be improved, and patients' recovery times were reduced. HBOT has been used for several purposes, such as patient and wound care. HBOT facilitated the supply of oxygen to the biological body's cells. While HBOT has a variety of applications in dental care, it is most frequently utilized to prevent complications during radiation therapy. 9 This review aimed to discuss the potential effects of HBOT in orthodontic treatment in animal studies.

## Methods

### Research/Review Question

The first approach in undertaking a complete systematic review was to characterize the problem through an organized study. 10 Patient/Population, Intervention, Comparison, and Outcome format was one method for transforming a clinical trial into a review question. 10 11 The research question for this study referred to the following; P (Patient/population): Patients under orthodontic treatment, I (Intervention): Orthodontics treatment with HBOT, C (Comparison): Orthodontic treatment without HBOT, and O (Outcome): Efficacy of HBOT as an adjunctive method in orthodontic treatment.

### Inclusion/Exclusion Criteria

Dhammi and Haq noted that it was necessary to specify the studies to be included and those omitted while doing a systematic review. Generally, studies with a significant level of evidence were required. However, if the subject of the review was insufficient, it was prudent to incorporate research at a lower level. Additionally, the language in which the paper was written was critical. Although restricting the systematic review to articles written in English would influence the result, it might be desirable if translation services and other resources were limited. Furthermore, it was necessary to note the relevant research date of publication. Finally, it should be determined if just human research or human and animal studies will be included. 10

All relevant articles were included because of a lack of research on this issue. Both human and animal studies were included and categorized as gray articles (academic papers, including theses and dissertations, research and committee reports, government reports, conference papers, and ongoing research). All remaining publications' titles and abstracts were carefully reviewed to rule out those not meeting the inclusion criteria. Articles that did not examine the use of HBOT in orthodontic treatment were excluded.

### Systematic Literature Search

To discover papers relevant to the issue of this systematic review, a comprehensive search of the PubMed and Google Scholar databases was done. First, as many significant themes as possibly linked with this review topic should be identified using keywords/Medical Subject Headings terms (“orthodontic treatment”) and (“hyperbaric oxygen therapy”). Then, these keywords were merged using the Boolean operator (AND). Finally, no filters were applied to the PubMed and Google Scholar searches due to a lack of relevant evidence.

**Fig. 1:**
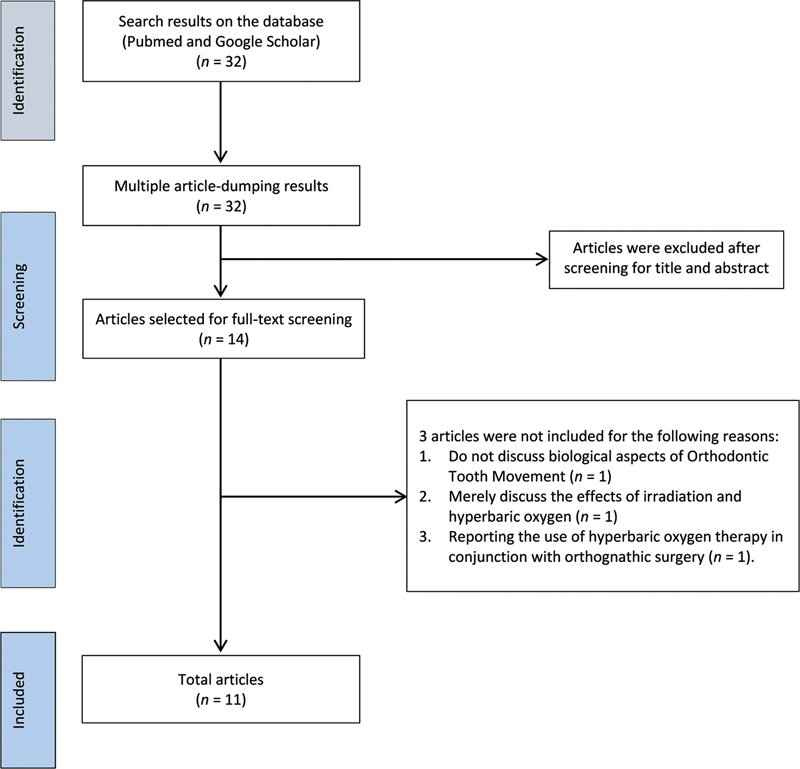
Preferred Reporting Items in Systematic Reviews and Meta-Analyses (PRISMA) flowchart.

The papers obtained in this search were examined individually, using the manuscripts' title, abstract, and full text to determine their eligibility. The references listed in the included papers were further reviewed to see whether any more published articles were overlooked during the database search. The flowchart in Fig. 1 depicts the study's exact flow and several Preferred Reporting Items in Systematic Reviews and Meta-Analysis items.

### Quality Assessment of the Included Articles

A quality evaluation or critical appraisal of the included articles was conducted to systematically assess and interpret the research's validity, findings, and significance. The internal and external validity of the studies was evaluated using the following criteria: descriptive bias, selection bias, measurement bias, analytic bias, and interpretation bias. The quality of all included papers was independently appraised by three reviewers (not including the authors). The quality assessment criteria of the National Institutes of Health (NIH) were used to evaluate if the quality was “good,” “fair,” or “poor.” 12

### Extraction of Data

After the papers' quality had been determined, the necessary data from each study should be extracted to answer the review question. Data were extracted and entered adequately into a well-designed spreadsheet. Data should be removed as feasible to ensure that nothing crucial was missed.

## Results

### Studies Included

The database search technique identified 32 potentially eligible references. Fourteen full-text publications were evaluated in their entirety after titles and abstracts were screened. Three papers were eliminated because they did not describe biological features of orthodontic tooth movement (OTM), discuss only irradiation and hyperbaric oxygen, and case report on the use of HBOT in orthognathic surgery. Eleven manuscripts were included in the literature review results ( Table 1 ).

**Table 1:** Literature research results

No	Author(s) and year	Title	Method	*n*	Objective	Result	Conclusion	Biased examination
1	Tuncay et al (1994) 16	Oxygen Tension Regulates Osteoblast Function	True experimental in vitro study	Eight osteoblast-enriched cultures from embryonic Sprague-Dawley rats aged 19 to 21 days	The purpose of this study was to determine the effects of ambient hypoxia and hyperoxia on osteoblast function in vitro by monitoring the media pH, pO _2_ , pCO _2_ , cell proliferation, alkaline phosphatase (AP) activity, and collagen synthesis	Low ambient oxygen levels promoted cell proliferation while decreasing AP activity, collagen production, culture pO _2_ , and PCO _2_ levels. Hyperoxia slowed cell development and increased AP activity, collagen formation, and the partial pressures of O _2_ and CO _2_	Changes in the ambient oxygen tension may have an effect on the osteoblastic function. Changes in oxygen availability activated the remodeling mechanism	Fair
2	Gokce et al (2008) 23	Effects of Hyperbaric Oxygen during Experimental Tooth Movement	True experimental	Twenty-four Sprague-Dawley rats	The purpose of this study was to determine the effect of hyperbaric oxygen on osteo regeneration following orthodontic tooth movement	HBOT increased the amount and density of trabecular bone while decreasing trabecular separation	HBOT promoted osteoblastic activity while inhibiting osteoclastic activity	Good
3	Inokuchi et al (2010) 24	The Effects of Hyperbaric Oxygen on Tooth Movement Into the Regenerated Area After Distraction Osteogenesis	True experimental	Ten dogs	The purpose of this study was to ascertain the effect of hyperbaric oxygen on newly formed bone in distracted areas surrounding the root of a moving tooth histologically and radiographically	Less trabecular bone density in the HBOT group compared with the control group. Bone and blood vessels grew on the tension side of the moving tooth in the HBOT group. The HBOT group's regenerated bone structure was larger and more active than the control group's	HBOT encourages bone ossification and vascularization	Good
4	Salah and Eid (2010) 25	Effect of Hyperbaric Oxygen on Mobility of Orthodontically Treated Teeth	True experimental	Twenty orthodontic patients	The purpose of this study was to determine whether HBOT had any influence on tooth mobility during orthodontic therapy	Teeth mobility was dramatically reduced in a group that received HBOT versus those who did not get HBOT	During and following orthodontic treatment, hyperbaric oxygen therapy (HBOT) may be beneficial in minimizing tooth movement	Good
5	Jonathan et al (2015) 19	The Influence of Hyperbaric Oxygen Therapy on Osteocyte Cell Number on Pressure Side during Orthodontic Tooth Movement	True experimental	Forty-two guinea pigs	The purpose of this study was to determine the influence of HBOT on the amount of osteocytes present on the pressure side of orthodontic tooth movement	The number of osteocytes differed between the groups	HBOT increased the quantity of osteocytes on the pressure side during tooth movement	Good
6	Brahmanta and Soetjipto (2016) 20	The Expression of Collagen Type-I in the Tension Area of Orthodontic Tooth Movement with Adjuvant of Hyperbaric Oxygen Therapy	True experimental	Twenty four Cavia cobaya	The purpose of this study was to assess the effect of HBOT on type I collagen expression in the tension zone of orthodontic tooth movement	HBOT improved type I collagen expression in the tension area	HBOT increased type I collagen in the periodontal ligament tension area, suggesting it could expedite orthodontic tooth movement	Good
7	Brahmanta and Prameswari (2019) 21	VEGF Regulates Osteoblast Differentiation in Tension and Pressure Regions Orthodontic Tooth Movement Administered with Hyperbaric Oxygen Therapy	True experimental	Twenty-four male guinea pigs	To see if VEGF increased osteoblast development in stress and pressure zone during tooth movement	HBOT elevated VEGF and osteocalcin levels in all locations except pressure regions	HBOT has been demonstrated to dramatically increase the expression of VEGF and osteocalcin in the tension region relative to the pressure zone. VEGF was able to promote osteocalcin synthesis	Good
8	Prayogo et al (2020) 18	The Changes of Fibroblast and Periodontal Ligament Characteristics in Orthodontic Tooth Movement With Adjuvant HBOT and Propolis: A Study In Guinea Pigs	True experimental	Forty-two male guinea pigs	An attempt to prevent orthodontic relapse by comparing periodontal ligament width and fibroblast count in the tension area after propolis gel and HBOT application	HBOT combined with propolis gel significantly increased the width of the periodontal ligament and the number of fibroblasts in the tension site in all groups	HBOT and propolis gel both had an effect on the width of the periodontal ligament and the number of fibroblasts in the orthodontic relapse area	Good
9	Prameswari et al (2020) 42	The Influence of Hyperbaric Oxygen Therapy (HBOT) to Intercausal Relationship Between Blood Vessels, Osteoblast, and New Bone Formation during Maxillary Suture Expansion	True experimental	Eighteen male guinea pigs	Correlate the number of blood vessels and osteoblasts in the maxillary suture expansion area during HBOT, showing new bone formation	HBOT enhanced the amount of blood vessels, osteoblasts, and the production of new bone during maxillary suture extension. There was a definite correlation between the quantity of blood vessels and osteoblasts and the production of new bone during maxillary suture expansion	HBOT increased blood vessel density, osteoblast density, and new bone formation during maxillary suture extension	Poor
10	Prameswari et al (2020) 17	Osteogenesis and Osteoclast genesis Regulation of the Midpalatal Area after maxillary Suture Expansion Induced by Hyperbaric Oxygen Therapy (HBOT)	True experimental.	Eighteen male Cavia cobaya	To investigate the regulation of osteogenesis and osteoclast genesis via ALP, TRAF-6, and midpalatal region following maxillary suture widening induced by HBOT	The strongest correlations were observed between osteoblast and ALP, osteoblast and osteoclast, ALP and osteoclast, and ALP and midpalatal region	HBOT enhanced osteoclast and ALP expression in addition to osteoblasts and the midpalatal region, while decreasing TRAF-6 expression. Osteoblasts contribute in the interaction of endothelial cells and osteoclasts during osteogenesis, whereas ALP is engaged in the mineralization of the midpalatal bone matrix	Fair
11	Brahmanta et al (2021) 22	The Effect of Hyperbaric Oxygen 2.4 Absolute Atmospheres on Transforming Growth Factor-β and Matrix Metalloproteinase-8 Expression during Orthodontic Tooth Movement In Vivo	True experimental	Twenty-four males Cavia cobaya	To determine the efficacy of HBOT as an adjunctive treatment to expedite periodontal ligament remodeling during OTM	HBO enhanced MMP-8 and TGF-β expression in the pressure zone and tension zone, respectively	HBOT was able to expedite the process of periodontal ligament restoration	Good

Abbreviations: ALP, alkaline phosphatase; HBOT, hyperbaric oxygen therapy; MMP-8, matrix metalloproteinase-8; OTM, orthodontic tooth movement; pO _2_ , partial pressure of oxygen; pCO _2_ , partial pressure of carbon dioxide; TGF-β, transforming growth factor-β; TRAF-6, TNF receptor associated factor 6; VEGF, vascular endothelial growth factor.

### The Studies Characteristics

Eleven articles with various research methods, samples, parameters, and quality were selected and reviewed. Due to the heterogeneity of the listed articles, no meta-analysis was conducted. For example, *in vitro* true experimental study method was used in one study, while 10 other studies were based on *in vivo* true experimental. The quality of evidence of the studies was analyzed by three independent raters (not including the authors). As indicated in Table 1 , the NIH quality assessment tool determined that eight papers were of excellent quality, two articles were of fair quality, and one item was of low quality or had a high potential for bias.

## Discussion

OTM biomarkers are those associated with inflammation, bone resorption, cell death, bone deposition, and mineralization. They are interleukin (IL)-1, IL-6, IL-8, tumor necrosis factor (TNF)-β, colony, macrophage colony-stimulating factor (M-CSF), granulocyte-CSF, granulocyte-macrophage-CSF, vascular endothelial growth factor (VEGF), prostaglandin E, calcitonin gene-related peptide, substance P, RANK, RANKL, G, aspartate aminotransferase, and lactate dehydrogenase, osteoprotegerin, as well as alkaline phosphatase (AP). 13 14 15 These biomarkers provide insight into osteogenesis and osteoclast genesis molecular and cellular biology in response to orthodontic mechanical pressures. In addition, parameters or biomarkers used to analyze in the included articles comprised AP), 16 17 collagen synthesis, 16 osteoblast, 18 osteoclast, 17 osteocyte, 19 type I collagen, 20 VEGF, 21 osteocalcin, 21 fibroblast, 18 matrix metalloproteinase-8 (MMP-8), 22 transforming growth factor (TGF)-β, ^22^ partial pressure of oxygen (pO _2_ ), 16 partial pressure of carbon dioxide (pCO _2_ ), 16 trabecular bone density, 23 24 teeth mobility, 25 PDL width, 18 number of blood vessels, 18 and TNF receptor associated factor 6 (TRAF-6). 17 From this perspective, it can be concluded that some other biomarkers involved in the orthodontic biological process might be further investigated.

Only two of those available articles comprehensively discussed the effect of HBOT on OTM in the pressure-tension terminology. HBOT promoted VEGF in the tension site but not in the pressure site. 21 Brahmanta et al concluded that HBOT increased TGF-β in the tension site and MMP-8 in the pressure site. 22 Those events in the tension site represented osteogenesis, while those in the pressure site represented osteoclast genesis. The remaining articles focused on one aspect of OTM terminology (compressive or tensile) or the general aspect.

OTM was a process that incorporated pathologic and physiological responses to environmental pressures. As a result, relevant inflammatory mechanisms had to be taken into account. The applied forces caused instantaneous strains in the tooth-supporting tissues, which might be classified as compressive or tensile. 26 27 28 Blood was squeezed out and vessels collapsed in the compressed periodontal membrane, dilating on the tension side. Although bone resorbed upon regeneration of the collapsed vasculature, dilation of blood vessels on the tension side appeared to stimulate subsequent bone deposition. The pressure-tension concept could be useful in distinguishing the various mechanisms associated with OTM. 16

Greater orthodontic forces resulted in necrosis and hyalinization, 28 29 30 31 and tooth movement could only be accomplished at the expense of bone resorption and a hypoxic periodontal membrane. 27 32 On the other hand, lesser orthodontic forces were associated with minimal necrosis and hyalinization. 28 29 Compression of the periodontal membrane and adjacent alveolar bone by orthodontic forces might result in apoptosis, hyalinization, and eventual hypoxia. 27 31 33 Tuncay et al discovered that low ambient oxygen tension increased cell growth while decreasing AP activity collagen synthesis, whereas HBOT-induced hyperoxia suppressed cell growth while increasing AP activity collagen synthesis. 16

The predominant mediators of neoangiogenesis were hypoxia-inducible factor-1 and VEGF. 31 34 35 36 Periodontal structures, which experienced significant remodeling during OTM, seemed to be susceptible to changes in O _2_ supply. 16 Hypoxia, caused by a vascular impairment, had been identified as a considerable constraint in wound healing. Therefore, supplemental oxygenation to treat hypoxia might have a significant beneficial effect on wound healing. Along with its nutritional and antibacterial properties, O _2_ might promote angiogenesis, cell motility, and extracellular matrix synthesis. 37

Angiogenesis plays a vital role in the early stages of healing. VEGF was a potent angiogenic promoter at the wound site. *In vivo* , oxygen boosted VEGF protein expression in wounds and VEGF messenger ribonucleic acid expression in endothelial cells and macrophages. 38 39 Collagen deposition was critical for wound healing because it served as the revascularization and tissue remodeling matrix. O _2_ was necessary for several posttranslational phases in the synthesis of collagen. Improved wound oxygenation increased collagen synthesis and elastic modulus, with the most significant effect happening when wound oxygenation was increased above physiological levels by adding more O _2_ . 37 40 41

The potential use of HBOT in orthodontic treatment seemed evident and supported by significant scientific evidence. However, HBOT had significant potential effects in orthodontic therapy. Almost fewer have been studied in orthodontic patients. Among the included articles, only one article used humans in the study. Salah and Eid used 20 orthodontic patients to evaluate the effect of HBOT on tooth mobility during orthodontic treatment. 25 The other studies used animals (Sprague-Dawley rats, 16 23 dogs, 24 guinea pigs, 18 19 21 42 and Cavia cobaya). 17 20 22 HBOT's effects got more investigated during inactive phase of orthodontic treatment. Only two studies described using HBOT in the passive phase to prevent orthodontic relapse. Salah and Eid demonstrated that HBOT might be useful in reducing tooth mobility during and after orthodontic treatment. 25 According to Prayogo et al, the interaction of HBOT and propolis gel affected the PDL width and the number of fibroblasts responsible for reducing orthodontic relapse risk. 18

The periodontium comprises gingiva, PDL, alveolar bone, and cementum surrounding the tooth root. 43 Compared with the other components of the periodontium, gingival tissue was not investigated in the included articles to highlight the comprehensive interrelationship between orthodontics, periodontics, and HBOT. Unlike the bone and periodontal membrane, the gingiva did not remodel promptly in response to orthodontic mechanical pressures; gingival collagen fibers were compressed and retracted for a lengthy time. In the compressed gingiva, the number of elastic fibers increased considerably. Compressed gingiva resembled compressed rubber, which would revert to its original proportions once the compression force was removed. Orthodontic relapse occurred due to the prolonged elastic deformation of gingival fibers, which impaired the surrounding teeth. 44 To maintain appropriate tissue stability and avoid orthodontic relapse, an equilibrium between collagen catabolic and anabolic gingival fibers was necessary. 45 HBOT offers some potential effects to be investigated further in preventing orthodontic relapse.

## Conclusion

To summarize, the potential for HBOT use in orthodontic treatment appears reasonably obvious and is supported by substantial scientific evidence. HBOT effects parameters or biomarkers that reflect the clinical, molecular, and cellular biology of osteogenesis and osteoclast genesis in periodontal tissues in responding to orthodontic mechanical pressure, such as AP, collagen synthesis, osteoblast, osteoclast, osteocyte, type I collagen, VEGF, osteocalcin, fibroblast, MMP-8, TGF.-b, pO _2_ , pCO _2_ , trabecular bone density, teeth mobility, PDL width, number of blood vessels, and TRAF-6. HBOT initiates an inflammatory response followed by OTM events during active orthodontic treatment. However, HBOT also plays a role in the tissue healing process during passive treatment. Nonetheless, additional research should be conducted to establish the efficacy of HBOT in orthodontic treatment in human studies.
